# CAR-T cell therapy in nephrology

**DOI:** 10.1080/0886022X.2025.2594261

**Published:** 2025-12-03

**Authors:** Francesco Tondolo, Elisa Gessaroli, Federica Maritati, Irene Colombini, Marinela Shkjau, Gaetano La Manna, Giorgia Comai

**Affiliations:** ^a^ Nephrology, Dialysis and Kidney Transplant Unit, IRCCS-Azienda Ospedaliero-Universitaria di Bologna, Bologna, Italy; ^b^ Department of Medical and Surgical Sciences (DIMEC), Alma Mater Studiorum-University of Bologna, Bologna, Italy

**Keywords:** Cell therapy, chimeric antigen receptor T, immune-mediated kidney diseases, autoimmunity

## Abstract

In recent years, chimeric antigen receptor T-cell (CAR-T) therapy has emerged as a promising immunotherapeutic strategy beyond oncology, offering new treatment opportunities for immune-mediated kidney diseases. These conditions including systemic lupus erythematosus, ANCA-associated vasculitis, membranous nephropathy, and monoclonal gammopathy-related nephropathies are characterized by dysregulated B cell and plasma cell activity that often proves refractory to standard immunosuppression. CAR-T cells, engineered to target antigens such as CD19 or BCMA, enable potent and durable depletion of pathogenic lymphocyte subsets, with growing evidence of clinical efficacy in autoimmune settings. Recent clinical data suggest that CAR-T therapies can induce profound immunological remission, restore immune tolerance, and improve renal outcomes. Novel platforms such as chimeric autoantibody receptor (CAAR)-T cells and bispecific T-cell engagers (BiTEs) further refine antigen targeting and may offer scalable alternatives. Importantly, early studies also point to the potential use of these therapies in kidney transplantation, particularly desensitization therapy in highly sensitized patients and treatment of post-transplant lymphoproliferative disorders. Despite these advances, challenges remain regarding toxicity, patient selection, cost, and long-term safety. This review critically evaluates the current landscape of CAR-based therapies in nephrology, explores their immunopathological rationale, and outlines future directions for their integration into clinical practice.

## Introduction

Immune-mediated kidney diseases comprise a heterogeneous group of disorders characterized by dysregulated immune responses targeting renal structures. These include autoimmune glomerulopathies such as systemic lupus erythematosus (SLE) associated nephritis, ANCA-associated vasculitis (AAV), and idiopathic membranous nephropathy (MN), as well as plasma cell dyscrasias with renal involvement such as multiple myeloma (MM) and AL amyloidosis. Despite their diversity, these diseases often share a common pathogenic feature: the expansion of autoreactive or abnormal B-cell populations that contribute to inflammation, antibody-mediated damage, and progression to kidney failure.

In recent decades, major advances in immunosuppression, particularly the use of monoclonal antibodies targeting CD20 such as rituximab, have transformed the management of several of these conditions. In idiopathic MN, for example, rituximab has demonstrated high efficacy and durable remission in many patients and is now a cornerstone of therapy [1–4]. However, in other settings such as lupus nephritis, AAV, and light chain–associated renal disorders clinical responses to B-cell directed therapies remain variable [5–8]. Incomplete remission, disease relapse, and persistent production of pathogenic antibodies despite B-cell depletion highlight the limitations of current approaches.

These challenges have spurred interest in next-generation immunotherapies that can achieve deeper and more sustained modulation of the autoreactive B-cell compartment. Chimeric antigen receptor (CAR)-T cell therapy, originally developed for hematological malignancies, is now being explored in autoimmune and kidney diseases as a means of targeting disease-driving B cell or plasma cell clones with greater specificity and durability [9–11]. Complementary strategies such as chimeric autoantibody receptor (CAAR)-T cells and bispecific T-cell engagers (BiTEs) have also emerged, with the potential to refine antigen targeting and expand treatment access [12,13].

This review provides an overview of the immunological rationale for these cellular therapies in nephrology, with a focus on B cell–driven conditions that remain refractory to conventional treatment. Particular attention is given to kidney-specific considerations, where therapeutic efficacy is defined by renal endpoints such as reduction of proteinuria, stabilization or improvement of estimated glomerular filtration rate (eGFR), and delay in progression to kidney failure. Moreover, since patients with chronic kidney disease (CKD) represent a particularly vulnerable population, the risk of treatment-related complications, most notably acute kidney injury (AKI) in the setting of systemic inflammatory states, must also be carefully considered in the evaluation of novel treatments. Our work examines the evolving evidence for CAR-T and CAAR-T therapies, assess their safety and feasibility in renal patients, and position them within the broader landscape of B-cell targeted immunotherapies.

## Immunological mechanisms in kidney disease

The immune system is intimately involved in the development and progression of many kidney diseases [14]. Inflammation and injury within the kidney are orchestrated by both innate and adaptive immune mechanisms that converge on the glomerular and tubulointerstitial compartments.

Innate immune responses provide the first line of defense against pathogens and tissue injury and are mediated by myeloid cells such as neutrophils, macrophages, and dendritic cells, along with soluble mediators including cytokines and components of the complement cascade [15]. In the kidney, activated neutrophils can drive acute tissue injury through the release of proteases, reactive oxygen species, and the formation of neutrophil extracellular traps (NETs), which are implicated in the pathogenesis of AAV and lupus nephritis [16,17]. Macrophages and dendritic cells contribute to both injury and repair, depending on their activation state, while dysregulation of the complement system, particularly the alternative pathway, is central to diseases such as atypical hemolytic uremic syndrome and C3 glomerulopathy [18]. The sustained activation of innate immune components not only leads to direct cytotoxicity but also sets the stage for adaptive immune activation.

Adaptive immunity, mediated by T and B lymphocytes, plays a pivotal role in the propagation and chronicity of immune-mediated kidney injury. CD8+ cytotoxic T cells can induce tubular and glomerular injury by directly killing renal epithelial or endothelial cells presenting cognate antigens [19]. CD4+ T helper cells, particularly Th1 and Th17 subsets, produce pro-inflammatory cytokines such as interferon-gamma (IFN-γ) and interleukin (IL)-17, promoting leukocyte recruitment and local inflammation [20]. Moreover, increasing evidence indicates that regulatory T cell (Treg) dysfunction is a central immunological abnormality in autoimmune kidney diseases: insufficient number and impaired suppressive capacity of Tregs contribute to persistent intrarenal inflammation and progressive fibrosis. This pathogenic imbalance highlights the clinical need for therapeutic strategies capable of restoring Treg function and reestablishing durable immune tolerance in order to achieve long-term renal protection [21,22].

B cells contribute to kidney pathology through several mechanisms: they produce pathogenic autoantibodies (e.g., anti-PLA2R in membranous nephropathy or anti-dsDNA in lupus nephritis), serve as antigen-presenting cells to activate autoreactive T cells, and form ectopic germinal centers that sustain local immune activation [23]. In more severe or chronic conditions, aberrant B-cell activity can evolve into clonal expansions characteristic of monoclonal gammopathy of renal significance (MGRS), MM with renal involvement and light chain (AL) amyloidosis, in which nephrotoxic monoclonal immunoglobulin components accumulate within renal tissue, causing progressive damage [24]. This immunopathological continuum from polyclonal autoreactivity to clonal B-cell driven disease justifies the growing interest in therapies that target B cells or their precursors with greater precision. Among these, CAR-T cell therapy has emerged as a strategy to deplete autoreactive or nephrotoxic B cells in a highly specific and potentially durable manner, offering new perspectives in the treatment of immune-mediated kidney diseases.

## CAR-T cell therapy: mechanisms and comparison with current cell therapies and monoclonal antibodies

**Figure 1.**
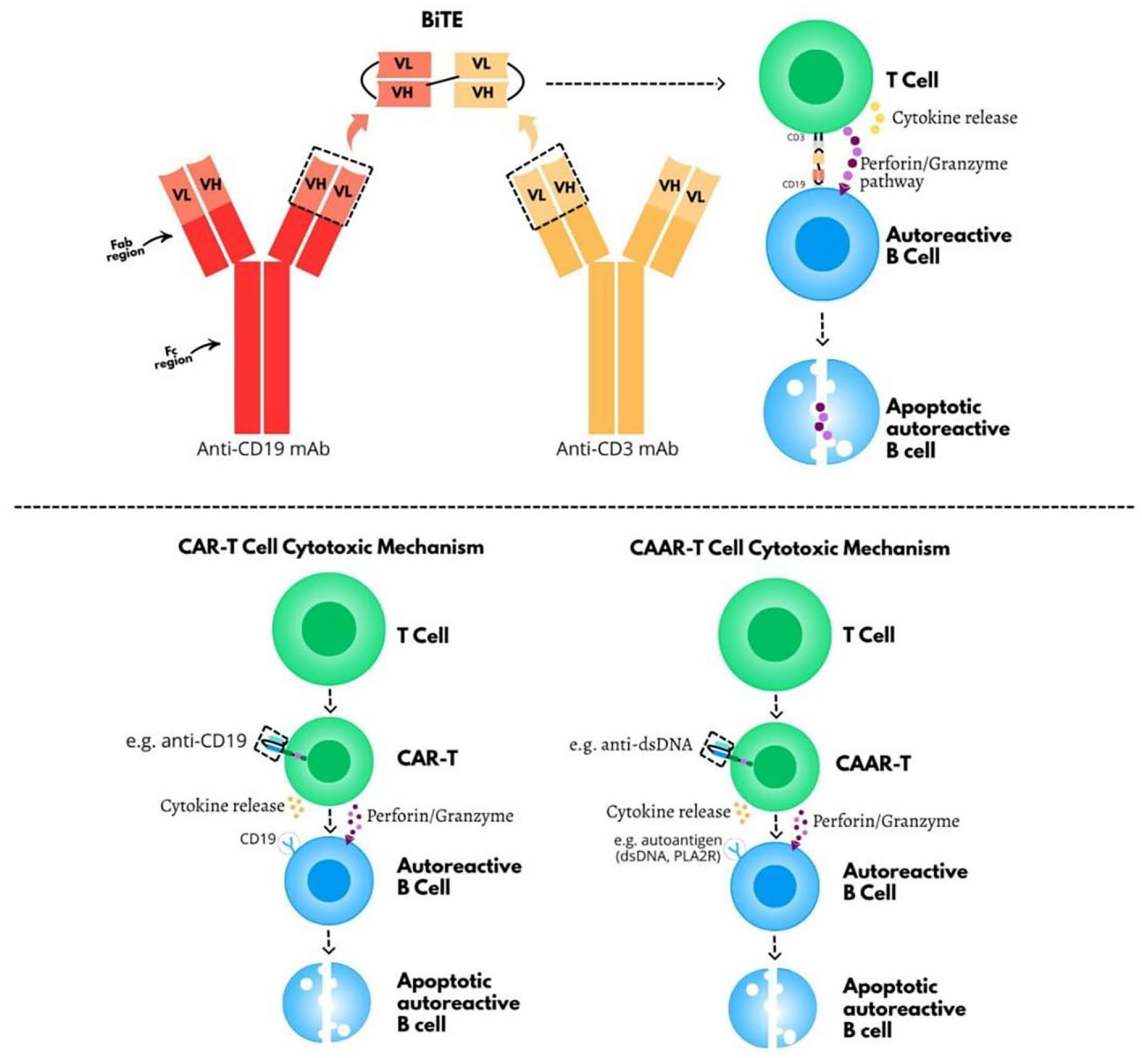
Comparison of BiTES and CAR-T cells. (A) Mechanisms of action of Bispecific T-cell engagers (BiTEs), composed of anti-CD3 and anti-CD19 scFv fragments. This interaction leads to T cell activation, cytokine release, and subsequent apoptosis of B cells *via* perforin/granzyme cytotoxic pathway. B) CAR-T cells, engineered to express a chimeric antigen receptor that directly recognizes antigens such as CD19 on B cells, and CAAR-T cells, which express a chimeric autoantibody receptor incorporating a disease-relevant autoantigen (e.g., PLA2R or dsDNA mimetic peptide). Both CAR-T and CAAR-T trigger T cell activation and cytotoxicity through cytokine release and perforin/granzyme pathways.

CAR-T cell therapy is based on the genetic reprogramming of autologous T lymphocytes typically *via* viral vectors to express synthetic receptors that target defined antigens on disease cells [25]. CAR constructs generally include an extracellular antigen-recognition domain (single-chain variable fragment), a hinge region, a transmembrane segment, and intracellular signaling domains responsible for T cell activation and co-stimulation [26] (Figure 1). Once infused into the patient, these engineered T cells can recognize and eliminate cells expressing the target antigen, such as CD19 or BCMA (B Cell Maturation Antigen), without requiring antigen processing or MHC presentation. This allows for efficient and sustained cytotoxicity even in immune-privileged or inflamed tissue compartments [10].

What distinguishes CAR-T therapy from other B-cell targeting approaches is its potential not only for direct depletion of pathogenic cell subsets but for broader and longer-lasting immune modulation. In contrast to monoclonal antibodies, which transiently deplete circulating B cells through mechanisms such as antibody-dependent cytotoxicity and complement activation, CAR-T cells can persist *in vivo*, expand clonally, and exert prolonged surveillance against reemerging pathogenic clones. Clinical data in refractory systemic lupus erythematosus and other autoimmune diseases have shown that CAR-T cells induce complete B-cell aplasia, eliminate circulating autoantibodies, and reshape the cytokine milieu, with reductions in BAFF and IFN-γ and restoration of regulatory T cell activity [9]. This reprogramming of the immune environment has been associated with durable remission and, in some cases, reversal of organ damage [27–29].

In a recent multicenter study of 15 patients with refractory autoimmune diseases (8 with SLE, 3 with idiopathic inflammatory myositis, 4 with systemic sclerosis), CD19-directed CAR-T therapy led to high response rates: all SLE patients achieved DORIS-defined remission, all myositis patients met major clinical response criteria, and systemic sclerosis patients had significant reductions in the EUSTAR activity index (median −4.2) and skin scores (median −9). Immunosuppression was discontinued in all cases. Adverse events were mostly mild, with only grade 1–2 cytokine release syndrome (CRS) and a single case of mild immune effector cell-associated neurotoxicity syndrome (ICANS) and pneumonia [29].

By comparison, monoclonal antibodies such as rituximab (anti-CD20) have demonstrated efficacy in several autoimmune kidney diseases, including MN, AAV, and lupus nephritis [1,2, 30,31]. However, their impact on disease activity can be partial and temporary in certain settings and diseases [7,32]. This is partly due to the inability of anti-CD20 agents to eliminate long-lived plasma cells, which lack CD20 expression but continue to produce pathogenic antibodies. Consequently, relapses are frequent despite apparent peripheral B-cell depletion. Dual-targeting approaches, such as combining anti-CD20 with agents targeting plasma cells or T cells, have been proposed to overcome this limitation, but increase the risk of immunosuppression and off-target effects. BiTEs such as teclistamab—which target CD3 on T cells and BCMA on plasma cells—may offer an alternative strategy. BiTEs are engineered bispecific molecules typically composed of two single-chain variable fragments (scFvs) connected by a flexible linker: one scFv binds to the CD3 receptor on T cells, while the other targets a specific antigen on the pathogenic cell (such as CD19 or BCMA), enabling direct T-cell activation and cytotoxic synapse formation independently of MHC presentation [33] (Figure 1). In a case series of four patients with severe autoimmune diseases including systemic sclerosis, Sjögren’s syndrome, idiopathic inflammatory myositis, and rheumatoid arthritis who had failed ≥5 prior immunosuppressants, Teclistamab induced marked clinical improvement, reduced autoantibody and immunoglobulin levels, and allowed glucocorticoid withdrawal in three patients. The treatment was well tolerated, with no neurotoxicity or myelotoxicity and only mild infections reported [34]. Similarly, a case report of a young woman with class II and V lupus nephritis showed resolution of proteinuria and normalization of serologies after five doses of Teclistamab [35]. More recently, a 76-year-old woman with refractory chronic immune thrombocytopenia and concomitant multiple myeloma achieved rapid and sustained platelet recovery and complete remission of both conditions after four doses of teclistamab [36]. While BiTEs have a shorter half-life and generally require continuous infusion or sustained delivery platforms, their immunomodulatory effects may be sufficient to achieve disease control in some settings. However, unlike CAR-T cells, they may not induce deep or lasting immune reshaping, and long-term efficacy in human autoimmune kidney disease remains unproven. Nonetheless, BiTEs represent a promising alternative in patients who are not candidates for cell-based therapy, or in resource-limited settings where CAR-T infrastructure is unavailable.

CAAR-T cells are a newer generation of engineered T cells that express a receptor containing an autoantigen instead of an scFv, allowing them to selectively target autoreactive B cells (Figure 1). In preclinical models of pemphigus vulgaris, CAAR-T cells targeting DSG3 effectively eliminated pathogenic B cells while sparing the rest of the immune system [37]. CAAR-T approaches may be especially suitable for autoimmune kidney diseases, where the antigenic target is well defined, such as PLA2R in MN or dsDNA in lupus nephritis.

Overall, the unique features of CAR-T therapy including MHC-independent targeting, *in vivo* expansion, memory formation, and immune reprogramming may confer advantages in selected autoimmune kidney diseases where current therapies fail to achieve lasting disease control. Nevertheless, their complexity, cost, and toxicity profile necessitate careful patient selection and further validation in controlled clinical trials.

## Applications of CAR-T cell therapy in kidney-related diseases

Recent international experience has confirmed the beneficial use of CAR-T technology in certain kidney-related diseases, leading to the development of pre-clinical/clinical studies and trials. Here we report the current knowledge of several pathologies with different type and grade of renal involvement by analyzing pre-clinical and clinical studies.

### Systemic lupus erythematosus and lupus nephritis

SLE is a chronic multi-organ inflammatory disease caused by an imbalanced B cell activity and subsequent production of autoantibodies [38,39]. Renal involvement is a common complication that confers a significantly high risk of morbidity and mortality. The development of new drugs targeting specific mechanisms involved in the pathogenesis of SLE has gained considerable attention in recent times and stems from two considerations: the persistence of B lymphocytes in immunological “niches” that are difficult to reach by monoclonal antibodies and the crucial role of plasma cells that do not express CD20 [40]. Currently, there is no definitive cure for SLE, and the available therapeutic options have limited efficacy in SLE patients, together with severe long-term side effects. A deep depletion of the B cell line responsible for producing autoantibodies would allow for a long-lasting remission, potentially representing a definitive cure for the disease through an immune system reset. For this purpose, CAR-T technology is based on the engineering of autologous T cells from SLE patients with CAR receptor targeting autoantibody-producing CD19+ B cells [9].

The first trial of *in vivo* use of CAR-T targeting CD19 was reported in 2019 by Kansal et al. [41]: animals treated with anti-CD19 CAR-T cells showed a rapid and deep reduction in native dsDNA auto-antibodies. Given the encouraging results in animal models, recent case series have confirmed that eradication of pathological B cells can lead to prolonged remission in both adult and pediatric patients [9,28], thus promoting these immune effector cell therapies to be a viable option for SLE population. A recent study by Mackensen et al. reported a deep B-cell depletion in five patients with SLEDAI-2K scores between 8 and 16 that were refractory to multiple lines of therapy, with a marked improvement of clinical symptoms and normalization of laboratory parameters (anti-dsDNA seroconversion, proteinuria improvement and complement factor normalization) within 3 months of CAR-T therapy [9]. Active clinical trials on the treatment of lupus nephritis are reported in Table 1. We reported the principal differences in immunological mechanisms and the heterogeneity of exclusion criteria regarding patients with kidney involvement. These variations are likely to complicate cross-trial comparisons of treatment efficacy.

**Table 1.** Ongoing CAR-T cell therapy trials in lupus nephritis and their renal exclusion criteria, including elevated serum creatinine levels, dialysis dependency, and specific histological classes of lupus nephritis.

Ongoing trials	Allocation	Intervention Model	Target	“Renal” Exclusion Criteria
NCT05085418	N/A	*Single* *Group Assignment*	CD19/BCMA Chimeric Antigen Receptor T Cells	Creatinine > 2.5 mg/dL
NCT06342960	Non Randomized	*Sequential Assignment*	Anti-CD19 Chimeric Antigen Receptor T-Cell	Rapidly progressive glomerulonephritis
NCT05938725	Non Randomized	*Sequential Assignment*	Anti-CD19 Chimeric Antigen Receptor T-Cell	Rapidly progressive glomerulonephritis
NCT06544330	N/A	*Single* *Group Assignment*	CD19 CAR-T Cell Therapy (SYNCAR-001) + Orthogonal IL-2 (STK-009)	Rapidly progressive glomerulonephritis End stage kidney disease requiring dialysis
NCT06429800	Non Randomized	*Sequential Assignment*	Allogeneic anti-CD19 Chimeric Antigen Receptor	Severe kidney disease (Class III, IV, V LN)
NCT06121297	N/A	*Single* *Group Assignment*	Autologous CD19-specific Chimeric Antigen Receptor T Cells	Uncontrolled kidney disease
NCT06316791	N/A	*Single* *Group Assignment*	Anti CD19 Cell Therapy	Dialysis patients
NCT06350110	N/A	*Single* *Group Assignment*	CAR T Cells Targeting BCMA/ CD19	Severe lupus nephritis (serum creatinine > 2.5 mg/dL or 221 μmol/L) within 8 weeks or subjects who need hemodialysis

### Membranous nephropathy

MN is the main cause of nephrotic syndrome in adults; in the past, the primary and secondary classifications were widely used, while in recent years a new classification has been proposed focusing on the target antigens driving the immune response [42]. The first kidney autoantigens identified in adults’ patients with MN were represented by phospholipase A2 receptor 1 (PLA2R1) and thrombospondin type-1 domain-containing protein 7 A (THSD7A), respectively in 70–80% and 2–5% of patient [43] The treatment of MN has also changed significantly in recent years: therapeutic regimens with CD20 targeted agents, such as rituximab, are used as first-line therapy and generally are well-tolerated. Rare episodes of resistance to monoclonal antibodies are reported in literature [44]. Therefore, the ideal treatment of this pathology should target the pathogenic autoantibody and/or the cells secreting these autoantibodies, sparing protective immunity: in this direction, an alternative therapy is represented by the possibility of eliminating autoreactive B cells using CAAR-T cells, a modification of CAR-T cells [45].

Specifically, chimeric autoantibody receptors (CAARs) incorporate a disease-relevant antigen fragment in place of the conventional single-chain variable fragment, enabling selective recognition of B-cell receptors (BCRs) on autoreactive B cells. This targeted interaction facilitates the specific elimination of pathogenic antibody-secreting cells (Figure 1).

In the setting of MN there is a clinical trial regarding the evaluation of the safety and efficacy of the BCMA/CD19 dual targeted CAR-T cell in participants with autoimmune kidney diseases (*NCT06285279, clinicaltrials.gov*).

A further novelty could be represented by the possibility of using immortalized natural killer (NK) cells: Seifert and colleagues report a pilot study where they analyzed the generation of CAAR natural killer cells and primary human CAAR-T cells expressing the immunodominant regions of MN antigens PLA2R1 and THSD7A; authors demonstrated that these cells eliminate anti-PLA2R1 and anti-THSD7A-secreting cells *in vitro* [43], but further *in vivo* studies are needed to demonstrate the potential therapeutic effects for MN.

### Anti-neutrophil cytoplasmic autoantibody (ANCA)-associated vasculitis (AAV)

Granulomatosis with polyangiitis (GPA), microscopic polyangiitis (MPA) and eosinophilic granulomatosis with polyangiitis (EGPA) are different types of AAV: all these conditions are characterized by different types of organs involvement, and renal involvement is usually represented by a rapidly progressive glomerulonephritis with poor outcomes. In the pathogenesis and in the progression of AAV, B cells play a fundamental role [46]. Centrality of B cells is underlined using rituximab as induction and maintenance therapy in these conditions, leading to favorable outcomes and high rates of disease remission. Multiple maintenance doses are often required to achieve such results [45]. In this setting, the most significant limitation of anti-CD20 monoclonal antibodies is the presence of CD20-negative plasma cells and the lack of rituximab activity on non-circulating B cells. Therefore, CAR-T cell therapy could be used for the complete elimination of self-reactive B cells in AAV.

In a recent pre-clinical mouse model of AAV, Lodka and colleagues described an effective depletion of B cells and plasmablasts by CD19 CAR-T cells, which also enhanced the Anti-Myeloperoxidase (MPO)-ANCA decline and, most importantly, protected from necrotizing crescentic glomerulonephritis [47]. Therefore, the purpose of using CAR-T in AAV is to obtain long-lasting drug-free remission: CAR-T cell therapy targeting CD19 can induce a deeper and more sustained depletion of B cells, in secondary lymphoid tissues as well. Schultze-Florey and colleagues reported on clinical case of a 64-years old man with refractory MPO AAV treated with the administration of anti-CD19 CAR-T cells: they demonstrated for the first time a decrease in anti-MPO titers and a reduction of proteinuria levels in a 6-week follow-up period [48]. Clinical trials in the setting of AAV are currently being conducted.

### Plasma cell dyscrasias and renal involvement

MM is the second most common hematological malignancy of adults in the Western world [49]. Renal involvement in MM may be variable: the main effect is attributable to the harmful impact of monoclonal free light chains (FLCs) on the glomeruli and renal tubules, leading to the development of light-chain cast nephropathy, immunoglobulin-related amyloidosis, monoclonal immunoglobulin deposition disease and other FLC-related conditions [45]. In this setting, CAR-T immunotherapy has brought substantial benefits in the prognosis of patients that are refractory to multiple lines of therapy or triple-class refractory [50]. In MM there are two CAR-T cell therapy products with U.S. Food and Drug Administration (FDA) approval: idecabtagene vicleucel (ide-cel) and ciltacabtagene autoleucel (cilta-cel) both against BCMA [49,50]. A study by Li et al. described an excellent hematological and renal response in all seven patients with relapsed or refractory MM (RRMM) and eGFR of 15–29 mL/min/1.73 m^2^ treated with CAR-T [51]. Successively, another study on 59 RRMM patients reported a significant improvement in renal function six months after therapy with anti-BCMA CAR-T in 18 patients with impaired renal function (eGFR <90 mL/min/1.73 m^2^) compared to 41 with eGFR >90 mL/min/1.73 m^2^ [52]. In a meta-analysis, Tariq and colleagues aim to explore the non-BCMA CAR-T options for the MM patients: GPRCD5D (G protein-coupled receptor, class C, group 5, member D), a protein found on the surface of multiple myeloma cells, can be and effective non-BCMA target for the treatment of MM [53].

Immunoglobulin light chain amyloidosis (AL amyloidosis) is a rare form of monoclonal plasma cell disorder; the proper diagnosis of this condition requires histologic confirmation of amyloid deposition and typing associated with the presence of monoclonal plasma cell disorder in the serum, urine or bone marrow [45]. Renal and cardiac involvement are the most involved organs, occurring in 70% and 60% of patients respectively [50]. Among the treatment approaches, the goal is represented by the elimination of the underlying plasma cell clone to stop fibril production and limit the progression of organ damage. In AL amyloidosis unresponsive to standard therapies, CAR-T cell therapy has shown encouraging preliminary results. Several case reports have associated the achievement of hematological response with improvement in renal function (creatinine decline, increased eGFR, decreased proteinuria) with third generation CD19 CAR-T therapy in the treatment of AL amyloidosis [54,55].

### CAR-T therapy in kidney transplant setting

Kidney Transplant (KT) is currently the best treatment for patients affected by end-stage kidney disease. KT improves clinical outcomes, health-related quality of life and prolongs patient survival compared to dialysis [56,57]. In recent years, the demand for organ transplants has exceeded available supply, resulting in longer waiting times and poorer clinical outcomes for patients with renal failure [58]. Despite the progressive expansion of allocation criteria, approximately one-third of patients on the transplant waitlist have preexisting anti-HLA antibodies that hinder successful matching, even when standard desensitization strategies such as B-cell depleting agents and plasmapheresis are used [59,60]. CAR-T cell therapy has garnered interest as a potential desensitization strategy, given the current lack of validated treatments capable of providing consistent and durable benefits [60]. Jarmi and colleagues used, for the first time, CAR-T cells to target and eliminate B cells in 6 high sensitized KT recipients with encouraging results on the inhibition of alloantibody production [59]. Moreover, in the transplant setting, post-transplant lymphoproliferative disorder (PTLD) remains one of the most serious complications in solid organ recipients, with a substantial impact on both graft and patient survival. Management strategies are heterogeneous—ranging from reduction of immunosuppression to single-agent anti-CD20 therapy (rituximab) or other forms of immunotherapy—and response rates consistently remain below 50% [61]. CAR-T may represent a more promising option of treatment of these conditions: at present, there are two types of CAR-T cells for relapsed/refractory diffuse large B-cell lymphoma (r/r DLBCL) in solid organ transplant. The management of immunosuppressive therapy is not univocally interpreted. Mamlouk and colleagues described the safety and efficacy of autologous CAR-T cell therapy in 3 KT recipients with r/r DLBCL [61]: in all 3 patients, authors withdrew immunosuppressive therapy before CAR-T infusion, but 2 patients demonstrated disease relapse after three and eight months. Oren and colleagues described a case of a 23-year-old woman with heart and kidney transplant who developed refractory PTLD treated with CAR-T cell therapy [62]: clinicians did not stop immunosuppressive therapy and reached favorable results. Further studies are needed to define optimal patient selection and to establish the most appropriate timing for discontinuing and resuming immunosuppressive therapy to minimize the risk of allograft rejection.

## Potential side effects of CAR-T cell therapy

Although CAR-T cell therapy offers significant clinical benefits, it is associated with a unique spectrum of adverse events, including CRS, ICANS, prolonged cytopenia, infections, and organ dysfunction. Therefore, prompt identification and appropriate management of these toxicities are essential to optimize outcomes.

Among these, CRS is the most prevalent adverse event following CAR-T cell infusion, with a widely variable incidence reported in 42–100% of patients and with severe forms (grade ≥3) occurring in 20–30%, depending on the CAR construct and disease type. CRS represents an acute, systemic inflammatory response triggered by the activation and proliferation of immune cells, particularly CAR-T cells [63]. Although the exact pathophysiology of CRS remains unclear, it is believed to result from the activation of both immune and non-immune cells after CAR-T cells recognize target antigens. This leads to a massive release of pro-inflammatory cytokines–a phenomenon referred to as a “cytokine storm”–which can result in significant tissue damage. Elevated serum levels of cytokines such asIL-6, IL-2, IL-8, IL-10, IFN-γ, and tumor necrosis factor-alpha (TNF-α) are commonly observed in affected patients. Clinical manifestations of CRS typically include fever, tachycardia, myalgias, capillary leak, hypotension, and hypoxia. In severe cases, it may progress to multiorgan failure, including cardiac dysfunction, acute respiratory distress syndrome (ARDS), neurologic toxicity, renal and hepatic impairment, and disseminated intravascular coagulation (DIC) [64]. CRS typically manifests within 1 to 14 days after CAR-T cell infusion, with a median onset between days 5–7, depending on the specific product, clinical trial design, and patient population [65]. Most cases resolve within 7 days, particularly with the use of targeted first-line treatment such as the IL-6 receptor antagonist tocilizumab, which has been approved by the FDA for severe or life-threatening CRS [66].

Alongside CRS, neurotoxicity is a frequently observed adverse effect following CAR-T cell therapy. The most well-characterized neurotoxicity in this setting is ICANS, which typically develops within a few days to weeks after infusion and often follows or overlaps with CRS [62–66]. ICANS is the second most common toxicity associated with CAR-T therapy. Incidence rates vary significantly across studies, influenced by the CAR-T cell construction, underly

ing malignancy, and patient characteristics. A systematic review of 75 clinical trials reported that approximately 27% of patients experienced ICANS of any grade, with around 10% developing severe neurotoxicity (grade ≥3) [67]. The clinical presentation of ICANS is highly variable and may range from mild symptoms—such as headache, fatigue, attention deficits, agitation, and word-finding difficulties (aphasia) to severe, life-threatening manifestations, including seizures, increased intracranial pressure, cerebral edema, and coma [63]. The pathophysiology remains incompletely understood, but current evidence points to immune-mediated endothelial activation and disruption of the blood–brain barrier, contributing to neuroinflammation and neurologic symptoms [68].

Ovarian toxicity following CAR-T therapy is primarily attributed to the lymphodepleting chemotherapy, particularly cyclophosphamide, which poses a risk of transient or permanent gonadal dysfunction. However, observational data suggest that menstrual function may recover post-treatment in a subset of patients, indicating that ovarian suppression is not always irreversible [69].

Another frequent adverse effect of CAR-T cell therapy is prolonged cytopenia. While transient cytopenia is commonly observed in the early post-infusion period, approximately 30–40% of patients experience grade ≥3 cytopenia persisting beyond 30 days. Cytopenias may include neutropenia, thrombocytopenia, and anemia, which are associated with an increased risk of complications, particularly opportunistic infections and bleeding events [70].

In addition to these well-known toxicities, AKI has emerged as a relevant, though less frequent, complication. AKI usually develops around 7 to 10 days post-infusion, often coinciding with CRS onset [71]. The reported incidence varies from 5% to 33%, influenced by patient characteristics and CAR-T constructs. Recent meta-analyses estimate that AKI occurs in approximately 17% to 22% of patients, with 4% to 10% requiring renal replacement therapy (RRT) [72,73]. The pathogenesis of AKI in this context is multifactorial and closely linked to systemic complications, while preexisting CKD further amplifies the risk of its occurrence. The main mechanism is prerenal injury driven by CRS: pro-inflammatory cytokines cause systemic vasodilation, increased capillary permeability, and hypotension, leading to reduced effective circulating volume. Prolonged or severe hemodynamic disturbances can lead to acute tubular necrosis (ATN) [73]. Cardiac complications related to CRS, such as cardiomyopathy and low-output shock, may worsen kidney perfusion, contributing to cardiorenal syndrome. Direct cytokine-mediated endothelial injury and intrarenal inflammation also contribute to tubular dysfunction. Less common causes include rhabdomyolysis (due to muscle swelling or compartment syndrome) and abdominal compartment syndrome from massive fluid accumulation [71]. Several clinical risk factors have been consistently associated with AKI development after CAR-T therapy. Higher grades of CRS (3 and 4) and ICU admission markers of severe systemic illness are strongly linked to increased AKI risk [73,74]. Baseline laboratory abnormalities such as reduced eGFR and elevated lactate dehydrogenase (LDH), a marker of tumor burden and systemic inflammation, also correlate with higher AKI risk [74,75]. Most AKI episodes are reversible with prompt supportive care, including intravenous fluid resuscitation and vasopressors to maintain renal perfusion and hemodynamic stability [75]. In severe cases, second-line immunomodulation, especially IL-6 receptor blockade with tocilizumab, may be necessary to reduce inflammation and prevent organ damage [76].

## Conclusion and future perspectives

CAR-T cell therapy represents a paradigm shift in the management of immune-mediated kidney diseases. By enabling deep, antigen-specific, and MHC-independent depletion of autoreactive or clonally expanded B cell and plasma cell populations, CAR-T therapy addresses key limitations of current immunosuppressive strategies such as incomplete remission, rapid relapse, and the persistence of long-lived pathogenic clones. Promising results in refractory lupus nephritis, AAV, MN and plasma cell dyscrasias highlight the biological and clinical rationale for expanding CAR-T cell use in nephrology. Engineered variants such as CAAR-T cells and BiTEs further enrich the therapeutic landscape by refining antigen selectivity and improving accessibility in patients who are not candidates for cell-based therapies.

However, these advancements must be weighed against significant challenges. First, the clinical application of CAR-T in nephrology remains in its infancy, and current evidence is largely derived from early-phase trials or case series with limited follow-up. Second, toxicities such as CRS, ICANS, and cytopenias, while manageable, require specialized infrastructure and expertise, limiting broad implementation. Third, the cost and logistical complexity of autologous CAR-T manufacturing may hinder scalability, especially in non-oncologic indications. Additionally, the long-term immunological consequences of deep B-cell aplasia remain uncertain, particularly in populations already at risk for infections and organ dysfunction.

Despite these barriers, the modular nature of CAR-T design offers opportunities for innovation tailored to nephrology. Antigen targets relevant to renal autoimmunity such as PLA2R or dsDNA could be incorporated into CAAR-T constructs, paving the way for highly selective B-cell depletion with reduced immunosuppression burden. Similarly, the combination of dual-targeting CAR-T cells (e.g., CD19/BCMA) may allow for simultaneous targeting of both naïve/memory B cells and antibody-secreting plasma cells, enhancing remission durability.

A particularly promising and underexplored frontier is the use of CAR-T and BiTEs in the kidney transplant setting, both in pre-transplant desensitization and post-transplant complication management. For highly sensitized candidates with anti-HLA antibodies, current desensitization protocols relying on plasmapheresis and anti-CD20 agents often yield suboptimal or transient effects. CAR-T therapies or BiTEs targeting memory B cells or long-lived plasma cells could provide a more definitive immunological reset, improving transplant eligibility and long-term graft outcomes. Preliminary experiences, though limited, suggest feasibility and alloantibody suppression with anti-CD19 CAR-T therapy. Moreover, CAR-T cells may hold therapeutic value in post-transplant lymphoproliferative disorders, offering a more targeted alternative to traditional immunochemotherapy, especially when rituximab fails.

Looking forward, several priorities must be addressed to enable responsible and effective integration of CAR-based immunotherapies into nephrology: (i) robust clinical trials in autoimmune and alloimmune kidney diseases to define efficacy, durability, and safety profiles; (ii) biomarker-guided patient selection to identify those most likely to benefit from CAR-T or BiTEs, potentially including immunophenotyping and autoantibody profiling; (iii) strategies to mitigate toxicity, including prophylactic measures, early intervention protocols, and exploration of less toxic CAR constructs or delivery systems (e.g., mRNA-based, transient CARs); (iv) ethical and economic frameworks to ensure equitable access, especially as applications expand beyond oncology.

In conclusion, while still experimental, CAR-T and related cellular immunotherapies hold the potential to transform the treatment paradigm for both autoimmune kidney diseases and alloimmune complications in transplantation. With thoughtful clinical translation, personalized immunomonitoring, and technological innovation, these therapies could one day shift the field from broad immunosuppression to durable, antigen-specific immune reprogramming.
